# Adherence to the healthy Nordic food index, dietary composition, and lifestyle among Swedish women

**DOI:** 10.3402/fnr.v59.26336

**Published:** 2015-03-11

**Authors:** Nina Roswall, Ulf Eriksson, Sven Sandin, Marie Löf, Anja Olsen, Guri Skeie, Hans-Olov Adami, Elisabete Weiderpass

**Affiliations:** 1 Danish Cancer Society Research Center, Copenhagen, Denmark; 2 Department of Medical Epidemiology and Biostatistics, Karolinska Institutet, Stockholm, Sweden; 3 Department of Biosciences and Nutrition, Karolinska Institutet, Stockholm, Sweden; 4 Department of Community Medicine, University of Tromsö, The Arctic University of Norway, Tromsö, Norway; 5 Department of Epidemiology, Harvard School of Public Health, Boston, MA, USA; 6 Genetic Epidemiology Group, Folkhälsan Research Center, Helsinki, Finland; 7 The Cancer Registry of Norway, Oslo, Norway

**Keywords:** healthy Nordic diet, dietary pattern, Nordic Nutrition Recommendations, cross-sectional study, lifestyle, adherence

## Abstract

**Background:**

Studies examining diet scores in relation to health outcomes are gaining ground. Thus, control for dietary factors not part of the score, and lifestyle associated with adherence, is required to allow for a causal interpretation of studies on diet scores and health outcomes.

**Objective:**

The study objective is to describe and investigate dietary composition, micronutrient density, lifestyle, socioeconomic factors, and adherence to the Nordic Nutrition Recommendations across groups defined by their level of adherence to a healthy Nordic food index (HNFI). The paper examines both dietary components included in the HNFI as well as dietary components, which are not part of the HNFI, to get a broad picture of the diet.

**Design:**

The study is cross-sectional and conducted in the Swedish Women's Lifestyle and Health cohort. We included 45,277 women, aged 29–49 years at baseline (1991–1992). The HNFI was defined by six items: wholegrain bread, oatmeal, apples/pears, cabbages, root vegetables and fish/shellfish, using data from a food frequency questionnaire. Proportions, means and standard deviations were calculated in the entire cohort and by adherence groups.

**Results:**

Women scoring high on the HNFI had a higher energy intake, compared to low adherers. They had a higher intake of fiber and a higher micronutrient density (components of the HNFI), but also a higher intake of items not included in the HNFI: red/processed meats, sweets, and potatoes. They were on average more physically active and less likely to smoke.

**Conclusions:**

Adherence to the HNFI was associated with a generally healthier lifestyle and a high intake of health-beneficial components. However, it was also associated with a higher energy intake and a higher intake of foods without proven health benefits. Therefore, future studies on the HNFI and health outcomes should take into account potential confounding of dietary and lifestyle factors associated with the HNFI.

Health-enhancing, regional diet patterns based on locally available foods have been proposed as a feasible and environmentally friendly approach to achieve better public health (1). It seems more likely that a dietary pattern exerts an effect on health, rather than individual dietary components (2). The interest in a health-promoting dietary pattern based on foods from the Nordic countries is increasing (3). Suggested items to include are, e.g., fish, cabbages, apples/pears, berries, root vegetables, wholegrain (rye, oats, barley), rapeseed oil, and low-fat dairy products (1, 4–8) , chosen based on their tradition as a food source in the Nordic countries and due to their health-promoting effects (1).

Observational studies have found an inverse association between Nordic diet scores and total mortality (9), colorectal cancer (in women only) (10), high-sensitivity C-reactive protein concentration (11), abdominal obesity (12), preeclampsia (13), excessive gestational weight gain (7), and having a low-weight baby (7). A general feature of all studies is that adherers to a healthy Nordic diet score also seem to have a generally healthier lifestyle than non-adherers (6, 7, 9, 10). The findings might thus be partly explained by residual confounding of a healthy lifestyle. However, several beneficial biological mechanisms of the included dietary components have been suggested (14–19) . Randomized intervention trials, where confounding is minimized, have found positive effects of a designed healthy, Nordic diet on a range of short-term health outcomes (4, 20–22) , suggesting direct health benefits in at-risk populations.

The aim of the study is to describe and investigate adherence to the healthy Nordic food index (HNFI) (9) in the Swedish Women's Lifestyle and Health (WLH) cohort, by assessing the distribution of the dietary components included in the index, and dietary components not part of the index, along with lifestyle, and socioeconomic factors. Furthermore, we compare dietary composition and nutrient density among adherence groups to the Nordic Nutrition Recommendations (NNR) (23).

## Materials and methods

### Study participants

The WLH cohort included women aged 29–49 at recruitment (1991–1992), as described previously (24). In brief, 96,000 women residing in the Uppsala Health Care Region were selected by random sampling, using the individual national registration number. They were sent an invitation and a questionnaire on diet, lifestyle, and socioeconomic factors. A total of 49,259 women returned a completed questionnaire. The study was approved by the regional Ethical Committee at Uppsala University, and the Ethical Committee at Karolinska Institutet, Stockholm. We excluded participants who emigrated (*n*=16), had an extreme energy intake, defined as outside the first (<1,840 kJ/d) and 99th (>12,232 kJ/d) percentiles (*n*=1,073), or lacked information on any included variable, except physical activity (*n*=2,893), leaving 45,277 women for the analyses.

### Food frequency questionnaire

The questionnaire included a 6 months food frequency questionnaire (FFQ), assessing frequency and quantity of 80 foods/beverages (25). When the FFQ was validated, reproducibility of a healthy dietary pattern was investigated by repeating the FFQ twice 1 year apart and showed a Spearman correlation coefficient of 0.63 (26). A validation study was conducted in relation to four 7-day records in 129 women. The correlation coefficient for the healthy dietary pattern was 0.59 (26). For total energy intake, another validation study found a mean (SD) intake of 5,585 kJ (1,579 kJ) in the total cohort using the FFQ and a corresponding number of 7,106 kJ (1,466 kJ) using the 7-day records – suggesting some underestimation in the FFQ (25). The FFQ covered on average 80% of the women's dietary intake, which is comparable to other FFQs (27).

### The healthy Nordic food index

The HNFI was developed by Olsen et al. (9), and we used their definition for calculation, including six food groups: wholegrain bread, oatmeal, apples/pears, cabbages, root vegetables, and fish/shellfish. In this study, cabbage was assessed by three questions (white/red cabbage; cauliflower; broccoli/Brussels sprouts), root vegetables by two (carrot; yellow turnip and beetroot), and fish/shellfish by four questions (Atlantic herring/herring/mackerel; salmon; cod/pollock/pike; shellfish). Further, women were asked about portion size (small/medium/large). Intake of apples/pears and oatmeal was assessed by one question on intake and one on portion size. Wholegrain bread was assessed by one FFQ-item and one question on slices per day/week.

We calculated median consumption of each food group in the cohort and computed the HNFI for each participant, by assigning a value of 1 if her consumption was above study median intake for that food group, and a value of 0 otherwise. This was based on the similar scoring system of the Mediterranean Diet Score (MDS) (28). For wholegrain bread and oatmeal, the median intake was 0, as more than 50% of the cohort did not consume these two components (Table 1). Thus, 1 point was instead given to all participants with any intake of wholegrain bread (42.0%) and oatmeal (40.3%). We summed the values for all food groups to obtain the HNFI. Consequently, the score varied between 0 and 6, with a higher score corresponding to a higher adherence. Low adherers were defined as those scoring 0–1 point, medium adherers as those scoring 2–3 points, and high adherers as those scoring 4–6 points.

**Table 1:** Intake of the food components included in the healthy Nordic food index (g/day) in the Women's Lifestyle and Health cohort

	Median	25th percentile	75th percentile
Whole grain bread	0	0	24.6
Oatmeal	0	0	16.4
Apples and pears	35.2	17.6	61.5
Cabbage^a^	8.1	2.8	14.5
Root vegetables^b^	11.0	5.3	25.6
Fish and shellfish^c^	20.7	13.7	30.5

a White and red cabbage, cauliflower, broccoli and Brussels sprouts.

b Carrot, yellow turnip and beetroot.

c Atlantic herring, herring, mackerel, salmon, cod, pollock, pike and shellfish.

### Energy, dietary composition, and nutrient intakes

Energy and nutrient intake of each participant was calculated using nutrient composition tables from the Swedish National Food Administration database (29). The supply of energy and nutrients from the food items included in the index was calculated as percentages of energy and nutrients from the total diet. Micronutrient density was calculated by dividing each participant's micronutrient intake with their energy intake in MJ.

Intake across adherence groups was compared to the 2012-edition of the NNR (23). For dietary composition, we used energy percentages (carbohydrates, protein, fat alcohol), for fiber and sodium absolute daily intakes in g/day, and for micronutrients (vitamins A, C, D, and E, folate, and iron) nutrient density per MJ were calculated.

### Dietary items not included in the HNFI

We included information on four food groups not part of the index: red/processed meat, sweets, and potatoes, defined as: red meat: beef, game, pork, ground meat and liver; processed meat: black sausage, liver paste, sandwich meat, and sausage; sweets: chocolate, fruit soup/porridge, ice cream, jam, sugar, cake, cookies, and Danish pastry; potatoes; boiled and fried.

### Individual characteristics

Information on self-reported body mass index (BMI), overall physical activity (5-point scale), smoking status (never/former/current), education, and age was included in the study.

### Statistical analyses

We used median values and 25th and 75th percentiles to describe intake of the HNFI components. We calculated means and standard deviations (variable BMI), or proportions (variables age, education, physical activity, smoking), of lifestyle, socioeconomic factors, and demographics for the entire cohort, and the three adherence groups. For age, education, BMI, physical activity, and smoking, we calculated the Jonckheere-Terpstra test (30) to test for a monotone dose-response relation over increasing adherence groups. The Jonckheere-Terpstra test is a non-parametric test robust to single outliers and does not rely on any assumptions of the data following a particular data distribution.

For components of the HNFI, additional dietary items, energy, dietary composition, and micronutrients, we calculated the intake by adherence group and plotted these in box-and-whisker plots, including joined medians to graphically illustrate development across adherence groups.

A total of 1,867 (4.1%) participants lacked information on physical activity. They were, however, included in this study, and missing information on physical activity is presented as a separate category in the results. For all other variables, participants with missing information were excluded.

All analyses were calculated using the statistical software SAS version 9.3 (SAS Institute Inc., Cary, NC, USA).

## Results

A total of 45,277 women were included in the final analyses. Distribution across adherence groups was: low (0–1 points): 20.8% of the cohort, medium (2–3 points): 46.1%, and high (4–6 points): 33.2%. The median intake of index components ranged from 0 g/day for wholegrain bread and oatmeal to 35.2 g/day for apples and pears (Table 1).

When we compared subject characteristics across adherence, there was a statistically significant test result in favor of a monotonic trend in all five variables, suggesting a somewhat older age, longer education, higher physical activity, and less smoking among the high compared to low adherers (Table 2).

**Table 2:** Lifestyle, socioeconomic, and demographic characteristics in the entire Women's Lifestyle and Health cohort, and by adherence to the healthy Nordic food index

	Healthy Nordic food index score				
		
	All *n*=45,277	0–1 *n*=9,395	2–3 *n*=20,891	4–6 *n*=14,991	*P* value from Jonckheere-Terpstra test^c^
Age, *n* (%)^a^					
29–35 years	14,159 (31)	3,279 (35)	6,408 (31)	4,472 (30)	<0.001
36–42 years	15,785 (35)	3,132 (33)	7,335 (35)	5,318 (36)	
43–49 years	15,333 (34)	2,984 (32)	7,148 (34)	5,201 (35)	
Education, years, *n* (%)^a^					
≤10	13,369 (30)	3,303 (35)	6,392 (31)	3,674 (25)	<0.001
11–13	17,740 (39)	3,757 (40)	8,159 (39)	5,824 (29)	
≥14	14,168 (31)	2,335 (25)	6,340 (30)	5,493 (37)	
Mean BMI (kg/m^2^) (SD)	23.5 (4)	23.3 (4)	23.5 (4)	23.6 (4)	<0.001
Physical activity level, *n* (%)^a^^b^					
1 (very low)	1,805 (4)	608 (7)	842 (4)	355 (2)	<0.001
2	4,647 (10)	1,110 (12)	2,219 (11)	1,318 (9)	
3	25,880 (57)	5,306 (57)	12,067 (58)	8,507 (57)	
4	7,409 (16)	1,272 (14)	3,256 (16)	2,881 (19)	
5 (very high)	3,669 (8)	615 (7)	1,627 (8)	1,427 (10)	
Missing	1,867 (4)	484 (5)	880 (4)	503 (3)	
Smoking status, *n* (%)^a^					
Never	18,692 (41)	3,270 (35)	8,408 (40)	7,014 (47)	<0.001
Past	13,413 (30)	2,540 (27)	6,167 (30)	4,706 (31)	
Current	13,172 (29)	3,585 (38)	6,316 (30)	3,271 (22)	

a Percentages calculated by columns.

b Percentages calculated in relation to women with information on physical activity, only.

c The *P* value tests for a monotone dose–response relation over increasing adherence groups.

SD=standard deviation; BMI=body mass index [body weight (kg)/height (meter)**2]; *n*=number of women; m=meter; kg=1000 g.

**Fig. 1:**
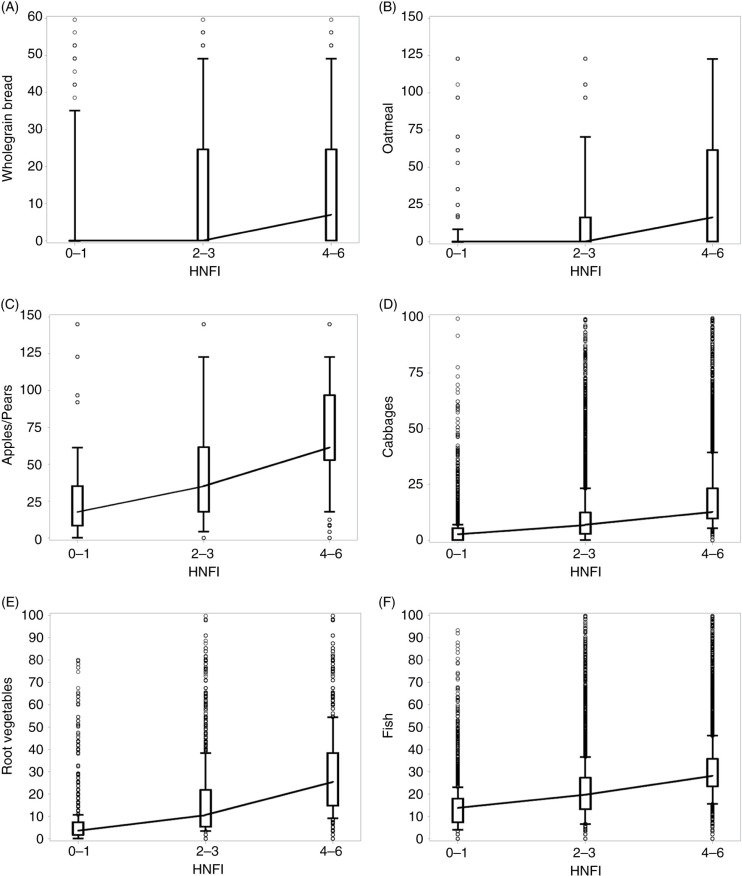
Box-plot distribution of intake of food groups (g/day) included in the healthy Nordic food index by HNFI adherence groups: low (0–1), medium (2–3), and high (4–6). Sub-panels, (A) Wholegrain bread, (B) oatmeal, (C) apples/pears, (D) cabbages, (E) root vegetables, (F) fish. HNFI=healthy Nordic food index. Boxes between 25th and 75th percentiles; joined medians; whiskers between 10th and 90th percentiles; individuals values as dots.

**Fig. 2:**
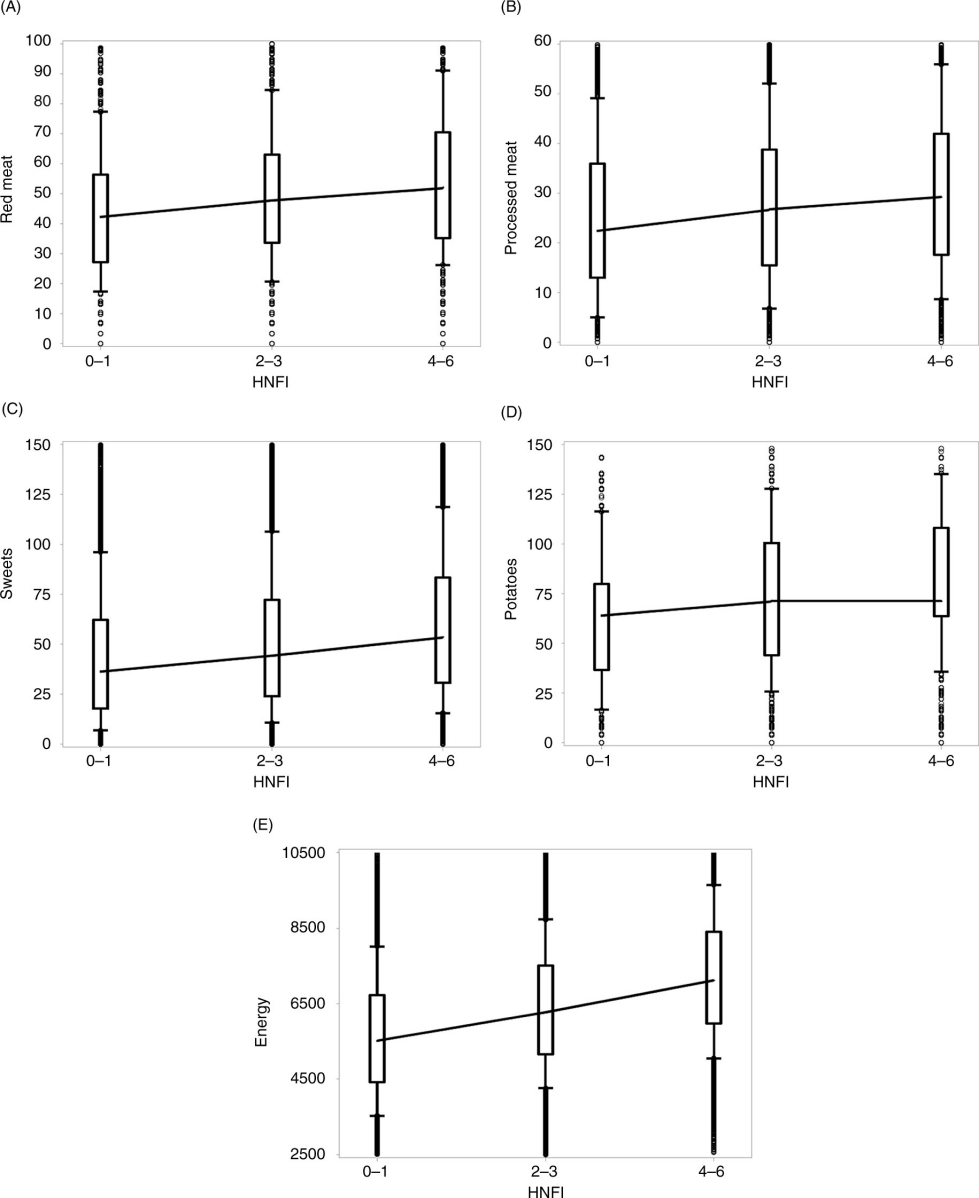
Dietary intake of food groups not included in the healthy Nordic food index, by HNFI adherence low (0–1), medium (2–3), and high (4–6). (A) Red meat, g/day, (B) processed meat, g/day, (C) sweets, g/day, (D) potatoes, g/day, (E) energy, KJ/day. HNFI=healthy Nordic food index; KJ=kilojoule. Boxes between 25th and 75th percentiles; joined medians; whiskers between 10th and 90th percentiles; individuals values as dots.

Figure 1 shows an increasing intake of all components of the HNFI with higher adherence. For most components, the increment is most pronounced between the medium and high adherers. However, we also found an increasing intake of dietary items not included in the index: red and processed meats, sweets and potatoes, and for energy intake. Here, the difference between the middle and high adherers was less pronounced, except for energy (Fig. 2).

**Fig. 3:**
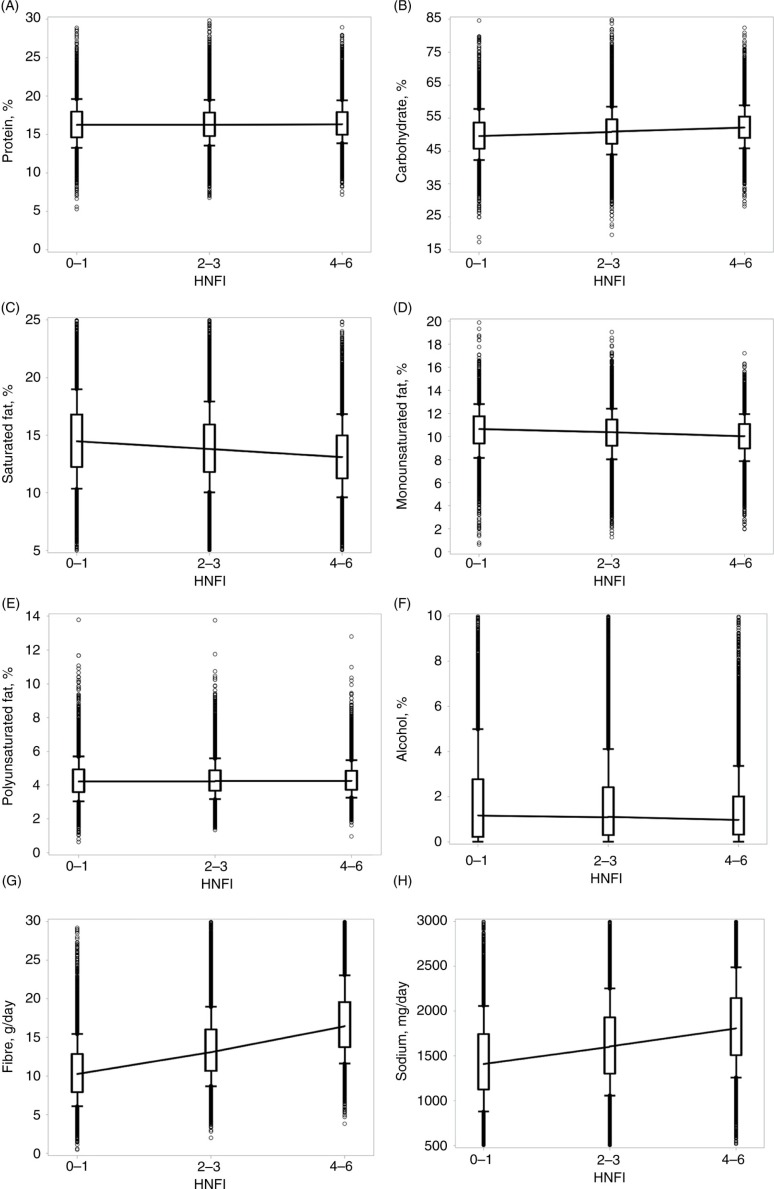
Dietary composition of the diet by energy percentage or g/day, by healthy Nordic food index (HNFI) adherence groups low (0–1), medium (2–3), and high (4–6). (A) protein, E%, (B) carbohydrates, E%, (C) saturated fat, E%, (D) monounsaturated fat, E%, (E) Polyunsaturated fat, E%, (F) alcohol, E%, (G) fiber, g/day, (H) sodium, mg/day. HNFI=healthy Nordic food index; E%=energy percentage. Boxes between 25th and 75th percentiles; joined medians; whiskers between 10th and 90th percentiles; individuals values as dots.

High adherers had a slightly higher intake of energy from carbohydrates [52 vs. 50 energy percentage (E%)], and a lower intake of alcohol (1.4 vs. 2.0 E%), saturated (13 vs. 15 E%), and monounsaturated fats (10 vs. 11 E%). E% from protein and polyunsaturated fat was similar across groups. High adherers consumed more fiber and sodium (Fig. 3) and had a more favorable ratio of unsaturated and omega-3-fatty acids to saturated fatty acids (results not shown). Compliance with the NNR was similar across adherence groups, with mean intakes within the recommendations for carbohydrates (45–60 E%), alcohol (<5 E%), protein (10–20 E%), monounsaturated fat (10–20 E%), and sodium (<2,400 mg/day), and none of the adherence groups reached the recommendations for polyunsaturated fat (5–10 E%), saturated fat (<10 E%), and dietary fiber (25–35 g/day) – the high adherers did, however, come closer than the low adherers (results not shown).

Those achieving a high index score also had a higher micronutrient density (Supplementary Fig. 1). Despite this, they only complied with the NNR for vitamins A and C, and iron, whereas the low adherers did so for vitamin A and iron (results not shown). The proportion of micronutrients coming from the HNFI was between 28.8% (vitamin A) and 11.4% (iron) among high adherers, compared to between 13.2% (vitamin C) and 6.0% (iron) among low adherers.

## Discussion

In this cohort of Swedish women, there was a broad variation in adherence to the HNFI. Those scoring highest on the index had a higher energy intake, with a slightly higher E% from carbohydrates, and a lower E% from alcohol and fat. They also had a more favorable ratio of unsaturated and omega-3 fatty acids to saturated fatty acids, and a higher intake of fiber and all included micronutrients. However, they also had a higher intake of red/processed meats, sweets, and potatoes. They were more physically active, and fewer were current smokers.

In nutritional epidemiology, the use of composite dietary pattern indices is gaining ground, as they may more fully capture dietary complexity and possible interactions between dietary components, which can be lost in reductionist, nutrient-based analyses (31–33) . It has been proposed to identify healthy eating patterns, and study their nutrient components, rather than taking nutrient intake as the starting point (31). Two previous studies have examined the dietary composition of a Nordic diet in a Finnish (6) and Norwegian (7) cohort, but there are no previous studies in Sweden. In the Finnish study, high adherence to the Nordic diet was associated with a higher intake of carbohydrates, fiber, iron, vitamins A, C, and D, and folate, and a lower intake of saturated fatty acids and alcohol (6). In the Norwegian cohort, high adherence was associated with a higher intake of energy, protein (E%), fiber, and several micronutrients, and a lower intake of fat (E%), and added sugars (E%). These results were similar to ours. In a study on MDS, higher adherence was associated with a higher intake of energy, carbohydrates, polyunsaturated fat, n-3 fatty acids, fiber, folate, vitamins C and E, and iron, a lower intake of saturated fat and no association with protein intake (34). These findings are similar to the studies on Nordic diet, suggesting that the mechanisms through which the two diet patterns assert their health-beneficial effects may be similar.

The Norwegian study also examined adherence to the Nordic diet and demographic characteristics and found that those with a high adherence were older and more educated than those with lower adherence (again findings matching the present study). Cohort studies on health outcomes have generally also found that those with a higher adherence have a healthier lifestyle (6, 7, 9, 10, 12). For the MDS, several studies have found that adherence is associated with more physical activity, less alcohol intake, less smoking (35–38) , increasing age (35, 37, 39), and higher socioeconomic status (36, 37). These findings are in accordance with the present study. Several studies found a lower BMI among high adherers to the MDS (40–44) , contrasting our finding of a seemingly higher BMI among those with the highest adherence to the index. However, the difference between groups was marginal. Still, health benefits of a Nordic food index or MDS in observational studies may arise from residual confounding, if analyses are not adjusted carefully for healthy lifestyle factors associated with, but not part of, the HNFI. The present study also examined associations between adherence to the HNFI and dietary items with no proven health-effects, such as potatoes (45), or even adverse health-effects such as red/processed meats (46, 47), and sweets (48). They were included as they represent an important part of total energy intake in the cohort. Here, we saw a direct association between adherence and intake. The association between adherence to the index and both a healthy lifestyle as well as unhealthy dietary factors suggests that residual confounding in future studies of the index and health outcomes may affect estimates in both directions, both strengthening and diluting the causal association between the HNFI and outcome. Studies on the index and health outcomes should thus carefully adjust for lifestyle as well as dietary factors outside of the index.

We found a higher compliance with the NNR among high adherers with regards to dietary composition (Fig. 3). For micronutrients, they only reached one more recommendation than low adheres; that of vitamin C (results not shown). The NNR is the main reference point for nutritional recommendations in the Nordic countries (23). The 2012 edition was not available when data were collected – changes between versions are, however, relatively minor (49). Two previous studies examined the composition of a Nordic diet and compared it to the NNR, and both found high compliance (5, 8). However, both were constructed diets, developed specifically for intervention studies, and direct comparison is thus not meaningful. They examined intake with full adherence to a diet constructed with the NNR in mind, whereas our study defined high adherers as those scoring 4–6 points, and our HNFI was not designed to capture the NNR.

The HNFI was originally designed for a Danish cohort (9), and adapted to this study by using the WLH FFQ to capture intake of the same food items. However, a study on intake of Nordic dietary components in Europe found a higher intake of apples/pears, cabbages, root vegetables, fish, and dark bread in Denmark compared to Sweden – five of the six components included in the index. In contrast, Swedish participants had a much higher intake of berries (50), suggesting that a Swedish-derived HNFI could have included berries, and may have found different results. However, the WLH FFQ did not ask about berries, but only fruit porridge and jam. In order to accommodate the geographical difference in bread intake, the HNFI was adapted to consider wholegrain bread rather than rye bread in the present study, but apart from this, the intention was to keep the index as similar as possible to the original index, in order to be able to reproduce the studies on mortality (9) and colorectal cancer (10). In general, intake of the index components was lower in the WLH cohort (Table 1), when comparing them to similar calculations in the Danish cohort (9, 10), suggesting some geographical variation in Nordic diet consumption. In WLH, less than half of the participants consumed two of the included foods: Oatmeal and wholegrain bread, suggesting that the index may not entirely capture high adherence to Nordic diet in the present cohort. For wholegrain bread, it may, however, be a result of limitations of the FFQ: We expect a large proportion of the wholegrain intake to come from wholegrain crispbread, but the FFQ did not separate between wholegrain and non-wholegrain crispbread.

The construction of the HNFI seems valid, as it draws upon the construction of the MDS (28), which is used extensively in epidemiological studies (51). We did not energy-adjust the dietary variables, as the purpose was to describe the HNFI, and how it is characterized, both by the variables included in the index, but also by variables that are not included in the index, such as energy intake. Furthermore, the HNFI was not created to measure energy intake, and the mechanisms through which we expect it to act are active ingredients such as micronutrients, fibers, and fat quality, rather than low energy content. From a public health perspective, the notion was to keep the index as simple as possible, so the interpretation of the results will be straightforward and easily communicated to the general public. We do, however, see a direct association between adherence to the HNFI and energy intake (Fig. 2). This could be explained by higher energy expenditure among high adherers, given their higher level of physical activity (Table 2). However, the FFQ was not designed to capture total energy intake, as it does not capture the entire diet (27), and the measurement of total energy intake in the study may therefore merely be considered a crude measure of the true energy intake. This also affects calculations on dietary composition and micronutrient density. The misclassification of energy intake should, however, be non-differential, and hence allow ranking of participants according to intake.

The strengths of the present study include the large sample size and detailed questionnaires. The validity and reproducibility of the FFQ has been tested previously (25, 26). In general, the validity of FFQs has been questioned (52, 53), as they use limited food item lists and approximated portion sizes, and are affected by each individual's perception of the questions and study context, as well as poor recollection, rendering participants easily affected by general knowledge on a healthy diet (27). The use of self-reported dietary data may induce bias, if there is differential misclassification; that is, the reporting depends on adherence to the index. With the use of a composite dietary index, the magnitude of bias may increase further; however, using the median as cut-off should minimize this, as people will be more likely to misreport within instead of between the two groups. However, it could also be an advantage, as the attention to health-enhancing effects of a Nordic diet had not yet surfaced when data were collected, hereby preventing participants from answering these questions favorably, based on a pre-conceived notion on healthy Nordic dietary items. Generalizability is hampered by the fact that the cohort includes women only and that the response-rate was fairly low (51.3%). This should not affect internal validity, but may complicate generalizability to the general Swedish population. The Jonckheere-Terpstra trend tests were statistically significant. However, this can almost be expected considering the sample size. Still, the data distributions showed important differences on an absolute level (Table 2).

In conclusion, a high score on the HNFI was associated with being a non-smoker, having a higher physical activity level, and a higher intake of fiber and micronutrients. However, high adherers also had a higher intake of dietary items with no proven health-beneficial effects: red/processed meats, potatoes, and sweets, and did not reach the NNR for several micronutrients. The present study serves as a basis for investigating the association between the HNFI and disease in the WLH cohort. Future studies examining associations between a HNFI and health outcomes should take into account potential confounding lifestyle and dietary factors not included in the index.

## Ethics

The study was approved by the regional Ethical Committee at Uppsala University, and the Ethical Committee at Karolinska Institutet, Stockholm.

## Supplementary Material

Adherence to the healthy Nordic food index, dietary composition, and lifestyle among Swedish women
