# Supplementary material for: Adherence to the healthy Nordic food index, dietary composition, and lifestyle among Swedish women
Source: Food Nutr Res. 2015 Mar 11;59:10.3402/fnr.v59.26336. doi: 10.3402/fnr.v59.26336 (PMC4359984; doi:10.3402/fnr.v59.26336)
Supplement: Adherence to the healthy Nordic food index, dietary composition, and lifestyle among Swedish women [file FNR-59-26336-s001.pdf]

Supplementary figure s1: Micronutrient density by healthy Nordic food index (HNFI) adherence-groups low (0-1), medium (2-3) and high (4-6) Sub-panels A) Vitamin E, mg/MJ; B) Vitamin D, µg/MJ; C) Vitamin C, mg/MJ; D) Vitamin A, Re/MJ; E) Folate, µg/MJ; F) Iron, mg/MJ

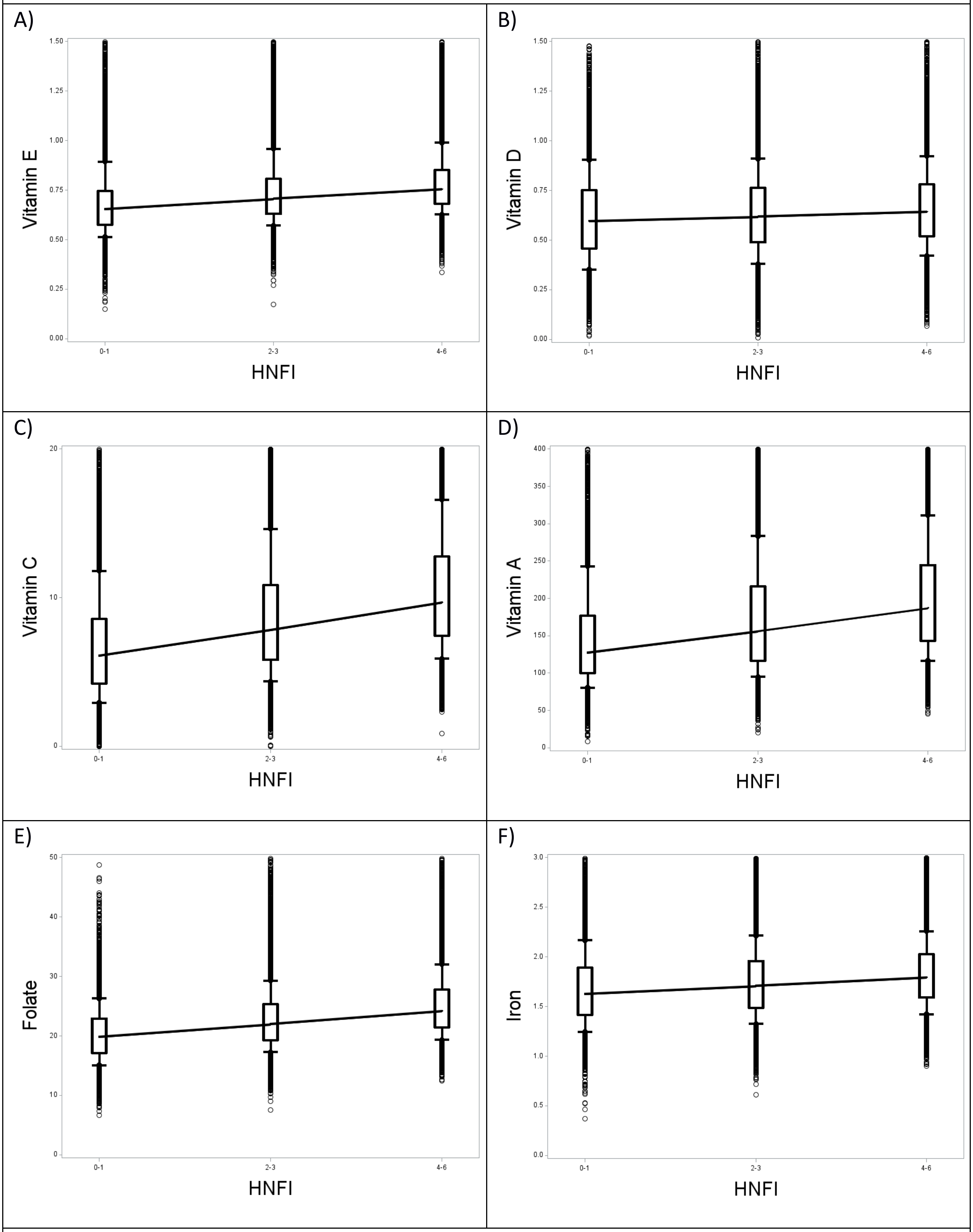

HNFI = Healthy Nordic food index

MJ = Megajoule

RE = Retinol Equivalents

Boxes between 25th and 75th percentiles. Joined medians. Whiskers between 10th and 90th percentiles. Individuals values as dots.
